# Ulnar variance as a predictor of persistent instability following Galeazzi fracture-dislocations

**DOI:** 10.1007/s10195-013-0266-7

**Published:** 2013-08-29

**Authors:** Richelle Takemoto, Michelle Sugi, Igor Immerman, Nirmal Tejwani, Kenneth A. Egol

**Affiliations:** 1 NYU Hospital for Joint Diseases, 301 East 17th Street Suite 1402, New York, NY 10003 USA; 2 Jamaica Hospital Medical Center, 8900 Van Wyck Expressway, New York, NY 11418 USA

**Keywords:** Galeazzi fracture, DRUJ, Radius fracture, Ulnar variance

## Abstract

**Background:**

We investigated the radiographic parameters that may predict distal radial ulnar joint (DRUJ) instability in surgically treated radial shaft fractures. In our clinical experience, there are no previously reported radiographic parameters that are universally predictive of DRUJ instability following radial shaft fracture.

**Materials and methods:**

Fifty consecutive patients, ages 20–79 years, with unilateral radial shaft fractures and possible associated DRUJ injury were retrospectively identified over a 5-year period. Distance from radial carpal joint (RCJ) to fracture proportional to radial shaft length, ulnar variance, and ulnar styloid fractures were correlated with DRUJ instability after surgical treatment.

**Results:**

Twenty patients had persistent DRUJ incongruence/instability following fracture fixation. As a proportion of radial length, the distance from the RCJ to the fracture line did not significantly differ between those with persistent DRUJ instability and those without (*p* = 0.34). The average initial ulnar variance was 5.5 mm (range 2–12 mm, SD = 3.2) in patients with DRUJ instability and 3.8 mm (range 0–11 mm, SD = 3.5) in patients without. Only 4/20 patients (20 %) with DRUJ instability had normal ulnar variance (−2 to +2 mm) versus 15/30 (50 %) patients without (*p* = 0.041).

**Conclusion:**

In the setting of a radial shaft fracture, ulnar variance greater or less than 2 mm was associated with a greater likelihood of DRUJ incongruence/instability following fracture fixation.

## Introduction

Galeazzi fractures are fractures of the radial shaft with concomitant distal radial ulnar joint (DRUJ) dislocation [1–3]. Previous studies have shown a relationship between the fracture distance from the radiocarpal joint and associated DRUJ injury [4]. One paper found that fractures 7.5 cm or more from the wrist are usually not associated with a DRUJ injury [4]. However, the absolute number of 7.5 cm does not take into account the variations in the size of the patient’s radius or where in relation to the radial bow a concomitant DRUJ dislocation will occur, as 7.5 cm is not proportionalized to variation in radial shaft length. Recently, Korompilias et al. [5] found that fractures located in the distal third of the radius were most likely to have DRUJ instability. Still, isolated distal radius fractures are far more common than Galeazzi fractures [6], decreasing the utility of using the distal third location to predict DRUJ instability. In our clinical experience, the currently reported parameters for DRUJ instability following radial shaft fracture are not universally predictive. The purpose of this retrospective study was to identify radiographic features of radial shaft fractures that could predict concomitant DRUJ dislocation after plate fixation of the radius.

## Materials and methods

This retrospective study was approved by our medical center’s institutional review board (IRB) and was performed in accordance with the ethical standards of the 1964 Declaration of Helsinki as revised in 2000. Our IRB approved the study without patient consent as it was purely retrospective and no identifiers were used. A review of a trauma database identified 66 consecutive patients by OTA or ICD-9 code who sustained diaphyseal fractures of the distal two-thirds of the radius and were treated surgically at three hospitals within an academic medical center from 2004 to 2009. All surgeries were performed by one of two trauma-fellowship trained attending physicians. Inclusion criteria were: patients over the age of 18 with a diaphyseal radial shaft fracture operatively treated at our medical center with complete radiographic follow-up of at least 6 weeks. Three patients were excluded because their index surgery was performed at an outside institution. Five patients were excluded because they were under the age of 18. Eight patients were excluded because of incomplete radiographic data and one patient died. Medical records and radiographs of the remaining 50 patients (76 %) were reviewed.

**Fig. 1:**
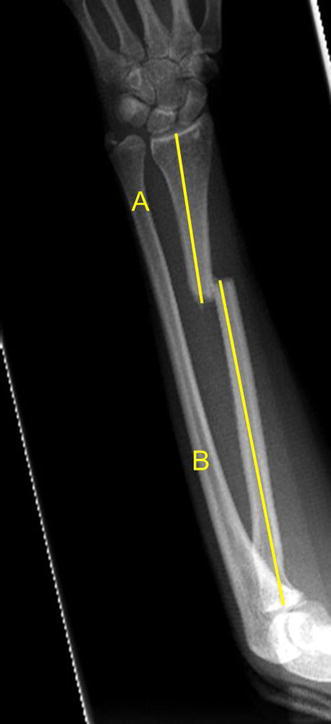
PA forearm radiograph: a ratio using the distance from the RCJ to the fracture divided by the entire length of radial shaft was then calculated to account for variations in the size of patient’s forearms (*a*/*a* + *b*)

**Fig. 2:**
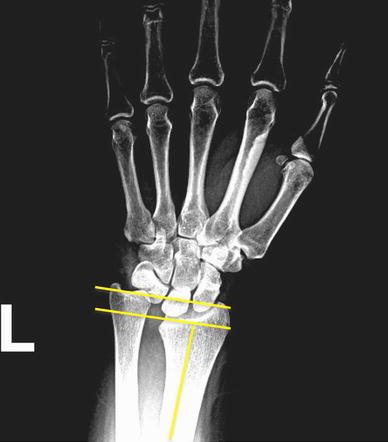
PA wrist radiograph: patient with a middle third radial shaft fracture, 4.27 mm ulnar positive with positive DRUJ instability. Ulnar variance measured with the digital calipers on the AP radiograph

Ten females and 40 males with an average age of 39 years (range 20–79 years) were followed for a mean of 7.1 months (range 6 weeks–1 year). Nineteen patients sustained multiple injuries to other extremities and 31 patients sustained an isolated upper extremity injury. Radiographic evaluation included AP and lateral forearm and wrist radiographs. Measurements of the radiographs were made using the digital caliper function on our electronic medical record system. To account for differences in the lengths of patient’s radii and location of the radial bow, a proportion of the distance from the RCJ to the radial fracture site was evaluated in relationship to the entire length of their radii. The length of the distal fracture segment was determined by measuring from the lunate facet (RCJ) of the injured radius to the most distal fracture site on the postero-anterior (PA) radiograph. The fracture location ratio was calculated by dividing the distance from the lunate facet to the fracture site by the length of the entire radial shaft on the PA forearm radiograph (Fig. 1). The injury ulnar variance was calculated on the PA wrist radiograph by measuring the absolute value of the difference between a line parallel to the intact ulnar articular surface with another line parallel to the ulnar aspect of the articular surface of the volar lip of the distal radius (Fig. 2). A single blinded investigator measured all radiographs at the time of data collection.

Twenty-seven patients underwent surgical fixation within 48 h of the injury. The remaining patients were treated on an average of 5 days (range 3–14 days) following injury. The radius was exposed using a volar (Henry) approach in 49 patients (98 %) and a dorsal (Thompson) approach in one patient (2 %).

Small fragment plates and screws were used for fracture repair in all cases (Synthes, Paoli, PA, Zimmer, Warsaw, IN, Smith and Nephew, Memphis, TN). The average number of screw holes on the plate was seven (range 6–10).

**Fig. 3:**
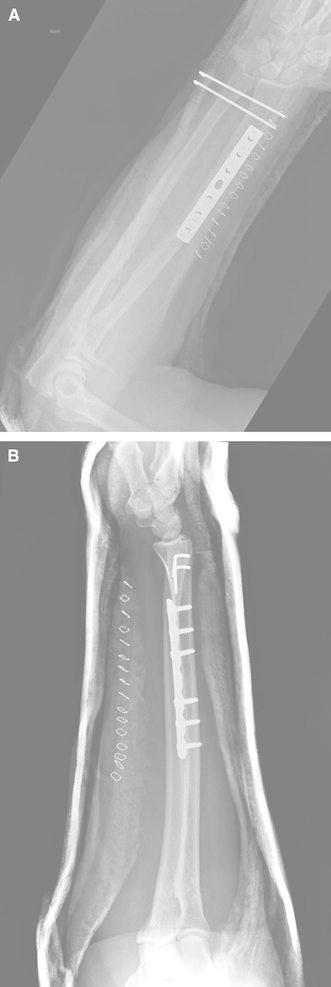
**a** AP forearm radiograph after surgical fixation and pinning of the DRUJ. **b** Lateral forearm radiograph after surgical fixation and pinning of the DRUJ

After surgical fixation of the radius, the DRUJ was assessed intra-operatively, compared to the contralateral extremity, and documents in the operative report by the surgeon. Persistent DRUJ incongruity/subluxation was determined to be present when, after fracture fixation there was (1) dorsal subluxation of the ulna on the lateral fluoroscopy image and (2) a difference in laxity compared to the contralateral side as determined by the treating surgeon, or (3) a dorsally prominent ulnar head in the setting of decreased supination. Persistent DRUJ incongruity/instability was found in 21/50 patients (42 %) after radius fracture fixation. Continued instability was treated with closed reduction and splinting in supination or manual reduction of the DRUJ followed by pinning of the DRUJ in neutral forearm rotation for 4–6 weeks (Fig. 3a, b) [4]. Ultimate treatment of the DRUJ was determined by surgeon preference as there is no universal protocol. Patients with a reduced and stable DRUJ intra-operatively were splinted in neutral and allowed gentle active forearm and wrist motion beginning 2 weeks post-operatively.

Patients were divided into two groups based on post-fixation status of DRUJ: stable (congruent, requiring no additional treatment) and unstable (incongruent, requiring further treatment). Student’s *t*-test was used to compare length of the distal fracture segment and location ratio, and Fisher’s exact test was used to compare ulnar variance, gender, incidence of polytrauma, and location of the fracture (SPSS Statistics 17.0, IBM Corporation, Somers, NY). Values of *p* ≤ 0.05 were considered significant. A multivariate regression analysis was performed for age, gender, polytrauma, ulnar variance, and location of fracture to identify factors associated with an unstable DRUJ following operative repair of radial shaft fractures.

## Results

A total of 50 patients with 50 radial shaft fractures were treated surgically. Radial fracture involved the distal third of the shaft in 24 patients (48 %) and the middle third in 26 patients (52 %). Thirty patients (6 females, 24 males) with an average age of 36 years old (range 20–70.7 years, SD = 13.8) had a stable DRUJ following surgical fixation. Twenty patients (4 females, 16 males) with an average age of 39.4 years old (range 19.8–78.7 years, SD = 15.1) were found to have an unstable DRUJ after surgical fixation. There were no significant differences between groups in age (*p* = 0.43), gender (*p* = 1), or percentage of patients with multiple extremity injuries (*p* = 0.39).

### Mean ulnar variance

The mean ulnar variance was 5.5 mm (median 4, range 2–12 mm, SD = 3.2) in patients with post-operative instability and 3.8 mm (median 2.5, range 0–11 mm, SD = 3.5) in patients without DRUJ instability. This was statistically significant (*p* = 0.037). No patient with post-operative DRUJ instability had pre-operative ulnar variance within −1 to +1 mm range. Preoperative ulnar variance within 1 mm of neutral was associated with a 100 % incidence of post-operative DRUJ stability. Based upon the work of Sanderson et al. [7] we assumed the normal range for ulnar variance to be between −2 and +2 mm. Of the 19 patients who presented with an ulnar variance within this range, 4 (21 %) had post-operative DRUJ instability, and 15 patients (79 %) had a stable DRUJ. In comparison, 16/31 (52 %) remaining patients (variance greater than ±2 mm) had post-operative DRUJ instability. This difference was significant (*p* = 0.041). Twenty-four patients had injury-induced ulnar variances within −3 to +3 mm, and 6 patients (25 %) had post-operative DRUJ instability. Conversely, 14/26 (54 %) remaining patients with ulnar variance out of the ±3 mm range had post-operative DRUJ instability. This difference was also significant (*p* = 0.048) (Table 1).

**Table 1:** Ulnar variance values correlated with incidence of post-operative instability

	Neutral	±1 mm	±2 mm	±3 mm	±4 to ±12 mm
Percent patients with DRUJ instability (%)	0	0	40	40	54

Ulnar variance was the only variable that was significantly associated with instability on regression analysis. For each 1 mm of variance away from neutral, there was a 26 % increase in the odds of having DRUJ instability with a 95 % CI (confidence interval). A separate subgroup analysis was performed for patients whose fractures were in the distal third of the radius. There were 8/20 (40 %) patients with fractures localized to the distal third of the radius in the unstable group, as compared to 16/30 (53 %) in the stable group. This association was not significant (*p* = 0.40).

However, within the subgroup of patients with fractures in the middle third of the radial shaft (12 unstable, 14 stable), injury-induced ulnar variance was a predictor of post-operative DRUJ instability. Using −2 to +2 mm as the normal ulnar variance range, one patient had a normal ulnar variance and an unstable DRUJ, versus eight patients with normal variance and post-operative DRUJ stability (*p* = 0.014). The remaining 17 patients had ulnar variances outside of the ±2 mm range, and of them, 11 (65 %) had DRUJ instability. These differences were significant (*p* = 0.014).

### Distance from RCJ to fracture

In the group with DRUJ instability, the average distance from the RCJ to the fracture was 89.0 mm (range 48.3–170 mm, SD 28) (Fig. 1). In the group without DRUJ instability the mean distance was 81.4 mm (range 27.3–134 mm, SD = 21.8). This difference was not significant (*p* = 0.31).

### Rettig criteria

The Rettig criteria of 7.5 cm from the RCJ was used as a cut-off value for predicting DRUJ instability [4]. A total of 17 patients had fractures within 7.5 cm, 10 patients were classified intra-operatively as stable and 7 patients were unstable. Thirty-three patients had fractures more than 7.5 cm from the RCJ of which 13 had an unstable DRUJ and 20 had a stable DRUJ. This difference was not significant (*p* = 0.24).

### Variation in forearm size

A ratio using the distance from the RCJ to the fracture divided by the entire length of radial shaft was then calculated to account for variations in the size of patient’s forearms (Fig. 1). In patients with DRUJ instability, the mean ratio was 0.37 (range 0.19–0.70, SD = 0.12). In patients without instability, the ratio was 0.34 (range 0.14–0.62, SD = 0.098). This difference was not significant (*p* = 0.34).

### Ulnar styloid fracture

There were four ulnar styloid fractures in the 20 patients (20 %) with DRUJ instability and 6 ulnar styloid fractures in the 30 (20 %) patients without DRUJ instability. The presence of an ulnar styloid fracture did not correlate with DRUJ instability.

### Complications

One patient with an unstable DRUJ splinted in supination sustained a post-operative peri-prosthetic fracture. One patient sustained an intra-operative radial artery laceration, which was repaired. Three patients resulted in nonunions, two of which had persistent DRUJ instability. Hardware was removed in two patients: one with DRUJ instability at 2 months and one without DRUJ instability at 2 years. One patient developed radioulnar synostosis, but elected not to have another surgery. Three patients with DRUJ instability later developed nerve compressions in the forearm, requiring late decompression more than 1 year after their index procedure. One developed a radial nerve compression, one developed a median nerve compression, and one developed an ulnar nerve compression. No patients without DRUJ instability developed late instability. No patient with initial DRUJ instability had persistent instability at latest follow-up.

## Discussion

Our results demonstrate that having ulnar variance of within −2 to +2 mm on the initial injury radiographs was associated with a 79 % likelihood of DRUJ stability and only 21 % chance of instability. Conversely, ulnar variance outside −2 to +2 mm was associated with 52 % chance of post-operative DRUJ stability. Thus, we found that ulnar variance greater or less than 2 mm on injury films is a predictor of DRUJ instability after radial shaft fixation. This data demonstrates that fractures less than 7.5 cm from the wrist joint do not correlate with DRUJ instability in the setting of a radial shaft fractures. We also found that radial shaft fractures with persistent DRUJ injury were seen more commonly in our series than previously reported, and that the location of the fracture in the distal third of the radius was not as strongly associated with DRUJ instability as previously reported.

This study also expands and amends the previously reported factors predictive of DRUJ instability. To our knowledge there are no clinical studies demonstrating a relationship between radial shortening and DRUJ stability. Our study demonstrates that shortening at the fracture site does not correlate with DRUJ stability. Ring et al. evaluated 36 patients with radius shaft fractures, of which nine patients (25 %) had concomitant DRUJ instability [4]. In Ring et al.’s study, the diagnosis of DRUJ injury was based on injury films of a measurement of 5 mm or greater of ulnar positivity as a surrogate measure of dislocation and not on intra-operative evaluation after radial shaft fixation as in our study [6]. Rettig et al. [4] described the distance from the RCJ to the fracture as the predictor of persistent DRUJ instability following fracture fixation. They suggested distinguishing isolated radius fractures with and without DRUJ injury on the basis of fracture location, with fractures 7.5 cm or greater from the lunate facet of the distal radius likely to be stable [4].

Our results differ from these two studies significantly. In the cohort of 40 patients studied by Rettig et al., variations in the size of radii were not accounted for. The cut-off value of 7.5 cm was an arbitrary value decided upon retrospectively after measurements of the fractures were made. The absolute value of 7.5 cm did not correlate with a specific percentage of the radius and variations in the size of radius were not evaluated. We measured the distance from the lunate facet as well as the total distance of the radius and created a ratio to account for differences in the size of patients. Using this approach, we found no association with the location of the fracture on the radius with instability of the DRUJ. Nineteen of 50 patients (38 %) had DRUJ instability after fixation of radius fractures. DRUJ instability after fixation of radial shaft fractures occurs more often than previous studies demonstrated [6].

In a recent study by Korompilias et al. [5], 40 out of 95 patients with radial shaft fractures were found to have persistent DRUJ instability following internal fixation of the radius. In their study, the authors found that the location of the fracture was a strong predictor of instability, with 37 cases of instability (54 %) out of 69 patients whose fractures were in the distal third of the radius [5]. Our results do not agree with their study, as only 33 % of the patients with fractures in the distal third had persistent DRUJ instability, and no statistically significant difference was noted. While it is possible that we failed to detect a difference due to the relatively low number of patients, the fact that a higher percentage of patients with the fracture in the middle third of the radius had instability strongly argues against this possibility. A potential explanation for the difference lies in the definition of the radius divided into thirds. Korompolias et al. [5] based their definition on the location of the radial bow, whereas we based it on a ratio of lengths determined by the distance to the fracture over the entire length of the radius. Fractures with the ratio of 0.33 or less are considered to be located in the distal third of the shaft. We believe that our method is more accurate, since the perceived location of the radial bow may change with rotation of the radiograph.

The only positive predictor of DRUJ instability after surgical fixation was the injury induced ulnar variance as seen on the injury films. Ulnar variance varies amongst the population [7]. Sanderson et al. [7] evaluated ulnar variance in over 1,000 cohorts and found that ulnar variance decreases with age. Another study showed that ulnar variance could differ between right and left hands in the same patient [8]. A 2002 study by Sonmez et al. [9] also demonstrated that ulnar variance can change dynamically with grip. Other studies have confirmed this normal ulnar variance in neutral rotation on PA radiographs [9]. Although studies agree that ulnar variance can vary in the population [7], the average variance is ±1 mm. We used ±2 mm as “normal variance” in our study to account for population variations in our cohort. Even so, we found a statistically significant difference in ulnar variance between the group with DRUJ instability and the group without instability.

Our study was limited by the retrospective data. It also is possible that some patients had spontaneous or assisted reductions before the initial radiographs, which would change the ulnar variance measured on the injury films. Lateral radiographs were difficult to obtain and varied with respect to rotation. These were evaluated, but not included in the study. The accuracy of restoring the normal anatomy, including the length and bow of the radius is essential for DRUJ stability. However, in higher energy injury mechanisms such as gun-shot wounds where bone loss occurs, the ulnar variance may not correlate with DRUJ stability after radial shaft fixation. We also only evaluated the plain radiographic parameters as predictors of DRUJ instability since this is the intent of the study, with no clinical outcomes. We did not use CT to evaluate the DRUJ. Though a CT would have been a reliable method of examining the DRUJ reduction, it was not standard of care in our community. Thus, this retrospective study could not have addressed this issue.

Our study questions previously accepted clinical data about DRUJ stability correlating with the location of the fracture on the radius or an absolute distance from the wrist joint. The DRUJ is often difficult to evaluate in these injuries and to date there are no radiographic markers to predict instability after a radius fracture. Our study demonstrates ulnar variance greater or less than 2 mm in the presence of an isolated radial shaft fracture is 79 % predictive of persistent DRUJ instability following fracture fixation. Thus, ulnar variance rather than absolute distance between the DRUJ and the fracture is the most reliable predictor of post-fixation persistent DRUJ instability. This knowledge allows surgeons to better pre-operatively counsel patients with fractures of the radial shaft.
